# Factors Influencing the Compliance of Pregnant Women with Iron and Folic Acid Supplementation in the Philippines: 2017 Philippine Demographic and Health Survey Analysis

**DOI:** 10.3390/nu13093060

**Published:** 2021-08-31

**Authors:** Eva Belingon Felipe-Dimog, Chia-Hung Yu, Chung-Han Ho, Fu-Wen Liang

**Affiliations:** 1 Department of Public Health, College of Health Sciences, Kaohsiung Medical University, No. 100, Shih-Chuan 1st Road, Sanmin District, Kaohsiung 807, Taiwan; u108863002@kmu.edu.tw; 2 Department of Nursing, Mountain Province State Polytechnic College, Bontoc 2616, Mountain Province, Philippines; 3 Department of Anesthesiology, Chi Mei Medical Center, No 901, Zhonghua Road, Yongkang District, Tainan City 710, Taiwan; dkntstar@hotmail.com; 4 Department of Medical Research, Chi Mei Medical Center, No 901, Zhonghua Road, Yongkang District, Tainan City 710, Taiwan; ho.c.hank@gmail.com; 5 Department of Medical Research, Kaohsiung Medical University Hospital, No.100, Tzyou 1st Road, Sanmin District, Kaohsiung 807, Taiwan

**Keywords:** compliance, iron and folic acid, pregnant, micronutrient supplement, Philippine

## Abstract

Anemia in pregnancy, which is a public health concern for most developing countries, is predominantly caused by iron deficiency. At least, 180 days of iron and folic acid (IFA) supplementation is recommended for pregnant women to mitigate anemia and its adverse effects. This study aimed to examine compliance with the recommendation of IFA supplementation and its underlying factors using the 2017 Philippine National Demographic and Health Survey data. The variables assessed included age, highest level of education, occupation, wealth index, ethnicity, religion, residence, number of pregnancies, time of first antenatal care (ANC) visit and number of ANC visits. Compliance with the recommendation of at least 180 days of IFA supplementation was the outcome variable. The study assessed 7983 women aged 15–49 years with a history of pregnancy. Of these participants, 25.8% complied with the IFA supplementation recommendation. Multiple logistic regression analysis showed that pregnant women of Islamic faith and non-Indigenous Muslim ethnicity were less likely to comply with the IFA supplementation recommendation. Being aged between 25 and 34 years, having better education and higher wealth status, rural residency, initiating ANC visits during the first trimester of pregnancy and having at least four ANC visits positively influenced compliance with IFA supplementation. The effect of residence on IFA adherence differed across the wealth classes. Strategies targeted at specific groups, such as religious minorities, poor urban residents, the less educated and young women, should be strengthened to encourage early and regular antenatal care visits for improving compliance.

## 1. Introduction

Approximately 40% of women have anemia globally, with more than half of the cases attributable to iron deficiency. Iron deficiency anemia is associated with adverse maternal and fetal health outcomes, including postpartum hemorrhage, low birth weight, preterm delivery and fetal growth restriction [1]. Deficiency to folic acid is associated with various maternal and fetal health conditions, including megaloblastic anemia in fetus, preeclampsia, abruptio placenta, spontaneous abortion, neural tube defects and adverse birth outcomes [2]. To combat anemia and improve feto-maternal health outcomes, the World Health Organization (WHO; Supplementary Table S1 includes a description of acronyms used) recommends iron and folic acid (IFA) supplementation for all pregnant women.

Guided by the WHO recommendation, the Philippine health authorities recommend daily IFA supplements containing 60 mg of elemental iron with 0.4 mg folic acid for at least 180 days for women during pregnancy and for up to three months postpartum [3,4]. The Philippine government has implemented programs to improve the accessibility and affordability of IFA [5]. The prevalence of anemia among pregnant women in Philippine has decreased from 38.3% in 2008 to 26.6% in 2018 [6] but remains a “moderate” public health problem [7]. The national survey data indicated that of the pregnant participants who had at least one antenatal care (ANC) visit, only 19% had taken the recommended 180 IFA tablets [8]. It was also found that a greater number of ANC visits was associated with better compliance with the recommendation [8]. However, this study did not investigate the relationships between the IFA recommendation compliance and the vital socio-demographic characteristics. Even though some studies reported factors associated with the sub-optimal compliance with the IFA recommendations [9,10], all of them were conducted on a small scale and limited to selected provinces. Therefore, to address barriers and leverage the factors that facilitate IFA compliance, in this study we aimed to estimate the compliance with the 180-day IFA supplementation among pregnant women in Philippine and to identify its associated factors using the data from the most recent available nationally representative survey.

## 2. Materials and Methods

### 2.1. Data and Sample

**Figure 1:**
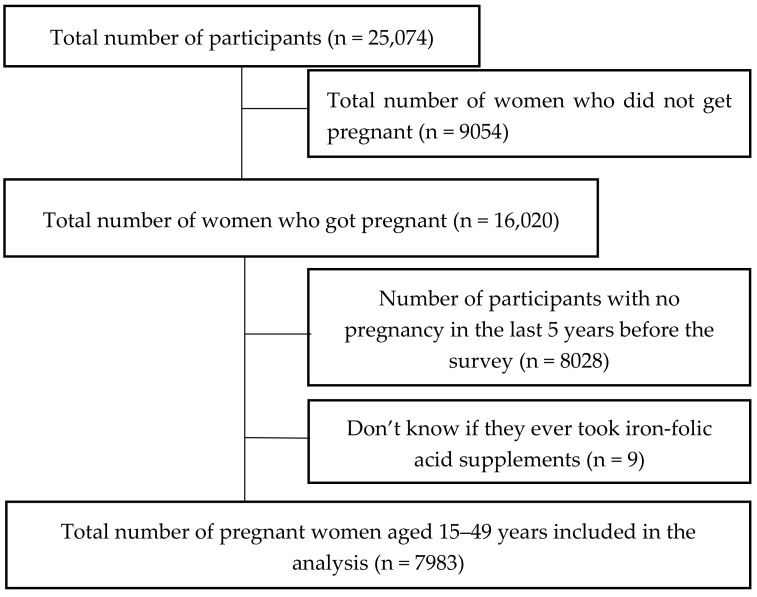
Flowchart for participant inclusion.

This study is a secondary analysis using data from the 2017 Philippine National Demographic and Health Survey (PNDHS) with the permission from the Demographic Health Survey Program. The PNDHS is a cross-sectional study using two-stage sampling techniques to obtain household samples from the entire country [11]. Further information about sampling techniques and survey methodology can be obtained from the PNDHS final report [11]. A total of 25,074 women aged 15–49 years completed the individual interviews during the survey, of which 16,020 had a history of pregnancy. Of the 16,020 interviewed participants, 7983 (49.8%) were included in the analysis for having been pregnant within five years before the survey (Figure 1).

### 2.2. Outcome Variable

The following question was asked to assess the intake of IFA tablets during pregnancy as part of the survey [11]: “During the entire pregnancy, for how many days did you take the IFA tablets?” The responses to this question were coded: 0 if IFA was not taken or was taken less than 180 days and 1 if IFA was taken for at least 180 days; this was the outcome variable of this study. Therefore, participants who took IFA for a minimum of 180 days during their recent pregnancy were categorized as the compliant group, and those who took IFA for fewer than 180 days were categorized as the non-compliant group.

### 2.3. Independent Variables

The independent variables used in this study were detailed in the PNDHS survey [11]. These independent variables were classified as follows: age of participants in years (15–24, 25–34 and 35–49); highest education attained (primary or below, secondary and higher); occupation (not working and working); wealth index (poor, middle and rich); ethnicity (non-Indigenous non-Muslim, non-Indigenous Muslim and others—based on the classification of the Philippine National Commission on Indigenous People in 2010) [12]; religion (Christianity, Islam and others or none); residence (urban and rural); number of pregnancies (1, 2, 3 and ≥4); timing of first ANC visit (do not know, first trimester and second or third trimester); and number of ANC visits (<4 ANC visits and ≥4 ANC visits).

### 2.4. Data Analysis

Descriptive statistics such as frequencies and percentages were used to describe the sociodemographic and antenatal characteristics of the participants. The Chi-squared test was used to examine differences in the distributions of independent variables between the compliant and the non-compliant group. Logistic regression analysis was conducted to examine the association between the characteristics of the participants and compliance with the IFA recommendation. The results were reported as crude and adjusted odds ratios with 95% confidence intervals. All statistical analyses were carried out using the Statistical Package for the Social Sciences (SPSS) version 20.0 (IBM SPSS Statistics for Windows, Version 20.0; IBM Corp., Armonk, NY, USA). Statistical significance was set at *p* < 0.05 (two-tailed).

## 3. Results

### 3.1. Characteristics of the Participants

This study involved 7983 women with a history of pregnancy. The sociodemographic and antenatal characteristics of the participants and their levels of compliance with the IFA recommendations are presented in Table 1. The majority of the participants were aged 25–34 years (47.7%), reported poor wealth (57.0%), completed secondary education (49.4%), had non-Indigenous non-Muslim ethnicity (68.1%), identified themselves as Christians (88.1%) and resided in rural areas (67.4%). In addition, two-thirds of the participants had their first ANC visit during the first trimester of pregnancy, while 4.0% could not remember their ANC visit. Most participants had four or more ANC visits (84.0%), and 4.1% never had an ANC visit. Of the 7983 participants, only 25.8% complied with the recommended minimum of 180 days of IFA supplementation. All the characteristics of interest, except residence, were significantly associated (*p* < 0.05) with compliance with the recommendations for IFA supplementation (*p* = 0.534). The proportion of participants who took IFA supplements for a minimum of 180 days was higher among those aged 25–34-years, the highly educated, the employed, the wealthy and Christians. More Islamic and non-Indigenous Muslim participants did not comply with the recommendation.

### 3.2. Associations between the Characteristics of Participants and IFA Supplementation

Logistic regression analysis indicated that age, highest educational attainment, wealth index, ethnicity, religion, residence, the timing of the first ANC visit and the number of ANC visits were significantly associated with compliance with IFA supplementation after controlling for potential confounders (Table 2). Pregnant women aged 25–34 years were 1.26 times more likely to take the IFA supplements (AOR: 1.26, 95% CI: 1.08–1.46) than those aged 15–24 years. Compliance was common among pregnant women with secondary and higher levels of education (AOR: 1.26, 95% CI: 1.07–1.49 for secondary; AOR: 1.72, 95% CI: 1.42–2.09 for higher). Regarding wealth status, rich participants were significantly more compliant (AOR: 1.40, 95% CI: 1.21–1.63) than their poor counterparts. In addition, participants who had their first ANC visit within the first trimester of pregnancy were 3.30 times more likely to take the supplements than those who had their first ANC visit later (AOR: 3.30, 95% CI: 2.85–3.83). Similarly, participants who had at least four ANC visits were 2.71 times more likely to be compliant than the others (AOR: 2.71, 95% CI: 2.08–3.52).

Ethnicity and religion negatively affect the level of compliance. Non-Indigenous non-Muslim and non-Indigenous Muslim participants were 15% and 45% less likely to be compliant than those of other ethnicities (AOR: 0.85, 95% CI: 0.75–0.97 for Non-Indigenous non-Muslim; AOR: 0.55, 95% CI: 0.31–0.95 for Non-Indigenous Muslim). Pregnant women of the Islam faith were also 64% less likely to be compliant than Christian participants (AOR: 0.36, 95% CI: 0.25–0.52).

To further determine whether the effect of residence on IFA supplementation compliance differed with wealth, the interaction between wealth index and residence was analyzed and found to be significant (*p* < 0.05). Poor rural residents were more compliant (AOR: 1.56, 95% CI: 1.27–1.92) than their poor urban counterparts. However, residence was not associated with IFA supplementation compliance for the middle class or the rich (AOR: 1.17, 95% CI: 0.90–1.51 and AOR: 1.22, 95% CI: 0.99–1.48, respectively) (Table 3).

## 4. Discussion

The findings of this study showed that only 25.8% of Filipino pregnant women complied with the 180-day IFA intake recommendation. On investigating the factors underlying this low compliance, the study found that Filipino pregnant women of Islam religion or non-Indigenous Muslim ethnicity were less likely to be compliant. On the other hand, Filipino pregnant women who were aged 25–34-years, had secondary and higher levels of education, had higher wealth index, were rural residents, had an early ANC visit initiation and had frequent ANC visits had increased odds of complying with the IFA recommendation. To the best of our knowledge, this is the first study to assess compliance with the recommendation of IFA supplementation for pregnant women and its associated factors using data from the most recent nationally representative survey. We further analyzed the effect of residence on compliance across different wealth classes and found that the poor urban residents were less compliant.

Compared to the findings of 2008 PNDHS, the ANC coverage was similar (96%), but the percentages of women who had their first ANC visit during the first trimester and 4 or more ANC visits were higher in our study: from 54% to 66% and 78% to 84%, respectively. Compliance with the 180-day recommendation for IFA supplementation was also higher in our study, but it remains low. The sub-optimal compliance with the recommendation of IFA supplementation among Filipino pregnant women in this study is consistent with the results of various studies [13,14,15,16]. Participants aged 25–34 years were significantly more compliant, which is probably because pregnant women in this age group are more responsible and have better health behaviors [17]. The better financial capacity enabling access to supplies and antenatal healthcare services and better access to health knowledge about anemia and effects of supplementation during pregnancy may explain the positive association between socioeconomic status and compliance. This is supported by the outcomes of studies conducted in Malawi and Ethiopia [15,16]. A higher wealth index of a mother has been reported as a significant determinant of compliance with the recommendation of supplementation [14,18,19]. Apart from improving wealth status, education facilitates the optimal utilization of information because of better understanding and mental capacity [20]. Higher levels of education, better wealth status and access to information are interrelated.

We found that Filipino pregnant women residing in rural areas were significantly more compliant than those in urban areas. This finding contradicts the result of a similar study conducted in Malawi. Malawian pregnant women from the urban areas were more compliant with iron supplementation [15]. We further found that the effect of residence on IFA compliance differed with the wealth index. Poor rural participants were more compliant than their poor urban counterparts; however, the significant association disappeared among the middle class and the rich. The better compliance of poor rural dwellers may be attributed to the various pro-poor health policies and programs implemented by the Philippine government. The government increased the number of healthcare providers to facilitate more effective and efficient health service delivery in rural and the most geographically isolated areas that have unserved and underserved communities through the Philippine Department of Health (PDOH) deployment programs, such as the Doctor to the Barrios (DTTB) program, the Registered Nurses for Health Enhancement and Local Service (RN HEALS) and the Rural Health Midwives Program [21]. Furthermore, the Barangay Health Workers (BHWs) and Barangay Nutrition Scholars (BNS) in the barangays (villages), together with the healthcare professionals, distribute IFA tablets and provide nutrition and health education to pregnant women [22]. The above-mentioned government programs are all community-based strategies to improve the access of poor rural dwellers to essential services. In addition, the WHO reported that urban poor dwellers are often neglected because health information of informal or illegal settlers, as well as the homeless population, is not collected [23]. This urban situation would mean that this study may have under- or overestimated IFA compliance among Filipino poor dwellers in urban areas. Regarding the antenatal characteristics, at least four ANC visits and the early ANC visits were positively associated with IFA supplementation compliance which is consistent with the reports of previous studies [13,14,24,25,26,27,28,29].

Some limitations of this study should be taken into consideration. First, no causal relationship can be inferred because the PNDHS is a cross-sectional survey. We could not determine the therapeutic effect of IFA on anemia or the potential side effects of long-term administration of iron preparations. This study merely depended on secondary data, which limited the elaboration of other significant responses such as healthcare providers’ attitude in IFA provision, length of waiting time for ANC services and availability of social support. In addition, recall bias was unavoidable in this study (although efforts were stated in the survey to minimize it). The conducted survey depended on verbal reports from the participants which limited a factual assessment of compliance to the supplementation. Nevertheless, this study reflects the most recent nationwide state of IFA supplementation compliance among Filipino pregnant women. Future research should qualitatively explore the influence of the practices of healthcare providers and the support of male partners in the utilization of ANC services by pregnant women, particularly IFA supplementation. Long-term follow-up of a birth cohort with the assessment of compliance with IFA supplementation during pregnancy and offspring development from birth to adulthood may also provide insights into causal relationships.

## 5. Conclusions

We found that approximately one in four Filipino pregnant women complied with the recommendation of IFA supplementation. Being aged 25–34-years, higher educational status, greater wealth, rural residence and early and frequent antenatal care visits were significantly associated with compliance with the IFA program. Pregnant women of Islamic religion or non-Indigenous Muslim ethnicity were less likely to be compliant. Therefore, strategies and health care programs targeted at specific groups, such as religious minorities, poor urban residents, the less educated and young women, should be developed or strengthened to encourage early and regular antenatal care visits and improve compliance. The results will help health authorities design tailor-fitted policies to facilitate compliance with IFA supplementation during pregnancy to improve maternal and fetal health outcomes.

## Figures and Tables

**Table 1:** The sociodemographic characteristics and selected survey responses by the number of days of iron-folic acid supplementation.

Variable/Categories	Total (N = 7983)	Non-Compliant Group (N = 5927)	Compliant Group (N = 2056)	*p*-Value
	*n* (%)	*n* (%)	*n* (%)	
Age (years)				<0.0001
15–24	1957 (24.5)	1530 (78.2)	427 (21.8)	
25–34	3804 (47.7)	2741 (72.1)	1063 (27.9)	
35–49	2222 (27.8)	1656 (74.5)	566 (25.5)	
Highest education attained				<0.0001
Primary or below	1634 (20.5)	1378 (84.3)	256 (15.7)	
Secondary	3942 (49.4)	2996 (76.0)	946 (24.0)	
Higher	2407 (30.1)	1553 (64.5)	854 (35.5)	
Occupation				<0.0001
Not working	4098 (51.3)	3132 (76.4)	966 (23.6)	
Working	3885 (48.7)	2795 (71.9)	1090 (28.1)	
Wealth Index				<0.0001
Poor	4554 (57.0)	3591 (78.9)	963 (21.1)	
Middle	1443 (18.1)	1091 (75.6)	352 (24.4)	
Rich	1986 (24.9)	1245 (62.7)	741 (37.3)	
Ethnicity				<0.0001
Non-Indigenous non-Muslim	5436 (68.1)	3941 (72.5)	1495 (27.5)	
Non-Indigenous Muslim	414 (5.2)	392 (94.7)	22 (5.3)	
Others	2133 (26.7)	1594 (74.7)	539 (25.3)	
Religion				<0.0001
Christianity	7031 (88.1)	5061 (72.0)	1970 (28.0)	
Islam	788 (9.9)	735 (93.3)	53 (6.7)	
Others or none	164 (2.0)	131 (79.9)	33 (20.1)	
Residence				0.534
Urban	2600 (32.6)	1919 (73.8)	681 (26.2)	
Rural	5383 (67.4)	4008 (74.5)	1375 (25.5)	
Number of pregnancies				<0.0001
1	1992 (25.0)	1422 (71.4)	570 (28.6)	
2	1992 (25.0)	1441 (72.3)	551 (27.7)	
3	1504 (18.8)	1111 (73.9)	393 (26.1)	
≥4	2495 (31.2)	1953 (78.3)	542 (21.7)	
Timing of first ANC visit				<0.0001
Don’t know	317 (4.0)	306 (96.5)	11 (3.5)	
First trimester	5290 (66.3)	3497 (66.1)	1793 (33.9)	
Second or third trimester	2376 (29.8)	2124 (89.4)	252 (10.6)	
Number of ANC visits				<0.0001
<4 ANC visit	1280 (16.0)	1201 (93.8)	79 (6.2)	
≥4 ANC visits	6703 (84.0)	4726 (70.5)	1977 (29.5)	

ANC = antenatal care

**Table 2:** Factors associated with compliance with iron-folic acid supplementation recommendations.

Variables/Categories	COR (95% CI)	AOR (95% CI)
Age		
15–24 ^R^	1.00	1.00
25–34	1.39 (1.22–1.58)	1.26 (1.08–1.46) **
35–49	1.22 (1.06–1.41)	1.20 (0.99–1.44)
Highest education attained		
Primary or below ^R^	1.00	1.00
Secondary	1.70 (1.46–1.98)	1.26 (1.07–1.49) **
Higher	2.96 (2.53–3.46)	1.72 (1.42–2.09) ***
Occupation		
Not working ^R^	1.00	1.00
Working	1.26 (1.14–1.40)	0.99 (0.88–1.10)
Wealth Index		
Poor ^R^	1.00	1.00
Middle	1.20 (1.05–1.38)	0.94 (0.81–1.10)
Rich	2.22 (1.98–2.49)	1.40 (1.21–1.63) ***
Ethnicity		
Non-Indigenous non-Muslim	1.12 (1.00–1.26)	0.85 (0.75–0.97) *
Non-Indigenous Muslim	0.17 (0.11–0.26)	0.55 (0.31–0.95) *
Others ^R^	1.00	1.00
Religion		
Christianity ^R^	1.00	1.00
Islam	0.19 (0.14–0.25)	0.36 (0.25–0.52) ***
Others or none	0.65 (0.44–0.95)	0.78 (0.52–1.17)
Residence		
Urban ^R^	1.00	1.00
Rural	0.98 (0.87–1.08)	1.30 (1.15–1.46) ***
Number of pregnancies		
1 ^R^	1.00	1.00
2	0.95 (0.83–1.10)	0.92 (0.79–1.07)
3	0.88 (0.76–1.03)	0.88 (0.74–1.05)
≥4	0.69 (0.60–0.79)	0.88 (0.74–1.06)
Timing of first ANC visit		
Don’t know	0.30 (0.16–0.56)	1.02 (0.53–1.98)
First trimester	4.32 (3.75–4.98)	3.30 (2.85–3.83) ***
Second or third trimester ^R^	1.00	1.00
Number of ANC Visit		
<4 ANC visit ^R^	1.00	1.00
≥4 ANC visits	6.36 (5.03–8.03)	2.71 (2.08–3.52) ***

ANC = antenatal care; COR = crude odds ratio; AOR = adjusted odds ratio; CI = confidence interval; ^R^ = reference; *** *p* < 0.0001; ** *p* < 0.01; * *p* < 0.05.

**Table 3:** The association between residence and iron-folic acid supplementation compliance across different wealth indexes.

Wealth Index	Non-Compliant Group	Compliant Group	AOR ^#^	95% CI
Poor, *n* = 4554				
Urban ^R^	716 (83.4)	142 (16.6)	1.00	
Rural	2875 (77.8)	821 (22.2)	1.56 *	1.27–1.92
Middle, *n* = 1443				
Urban ^R^	487 (77.8)	139 (22.2)	1.00	
Rural	604 (73.9)	213 (26.1)	1.17	0.90–1.51
Rich, *n* = 1986				
Urban ^R^	716 (64.2)	400 (35.8)	1.00	
Rural	529 (60.8)	341 (39.2)	1.22	0.99–1.48

^#^ adjusted by age, highest education attained, occupation, ethnicity, religion, number of pregnancies and timing of first ANC visit. * *p* < 0.05; ^R^ = reference; AOR = adjusted odds ratio; CI = confidence interval.

## Data Availability

Restrictions apply to the availability of these data. Data was obtained from the Demographic and Health Surveys (DHS) Program and are available from the first author with the permission of the DHS program.

## Supplementary Materials

The following are available online at https://www.mdpi.com/article/10.3390/nu13093060/s1, Table S1: List of acronyms used in this article.
