# Supplementary material for: Factors Influencing the Compliance of Pregnant Women with Iron and Folic Acid Supplementation in the Philippines: 2017 Philippine Demographic and Health Survey Analysis
Source: Nutrients. 2021 Aug 31;13(9):3060. doi: 10.3390/nu13093060 (PMC8468511; doi:10.3390/nu13093060)
Supplement: Supplementary file 1 [file nutrients-13-03060-s001.zip › nutrients-1353106-supplementary.pdf]

Supplementary Table S1. List of acronyms used in this article.

Acronyms	Explanation
ANC	antenatal care
BHW	Barangay Health Workers
BNS	Barangay Nutrition Scholars
DTTB	Doctor to the Barrios
IFA	iron and folic acid
IFAS	iron and folic acid supplementation
NNC	National Nutrition Council
PDOH	Philippine Department of Health
PNDHS	Philippine National Demographic and Health Survey
RNHEALS	Registered Nurses for Health Enhancement and Local Service
WHO	World Health Organization
